# Intratumor cholesteryl ester accumulation is associated with human breast cancer proliferation and aggressive potential: a molecular and clinicopathological study

**DOI:** 10.1186/s12885-015-1469-5

**Published:** 2015-06-09

**Authors:** David de Gonzalo-Calvo, Laura López-Vilaró, Laura Nasarre, Maitane Perez-Olabarria, Tania Vázquez, Daniel Escuin, Lina Badimon, Agusti Barnadas, Enrique Lerma, Vicenta Llorente-Cortés

**Affiliations:** 1 Cardiovascular Research Center, CSIC-ICCC, IIB-Sant Pau, Hospital de la Santa Creu i Sant Pau, Sant Antoni Mª Claret, 167 08025, Barcelona, Spain; 2 Department of Pathology, Hospital de la Santa Creu i Sant Pau, Barcelona, Spain; 3 Institut d’Investigacions Biomèdiques Sant Pau (IIB-Sant Pau), Barcelona, Spain; 4 Department of Medical Oncology, Hospital de la Santa Creu i Sant Pau, Barcelona, Spain; 5 Universitat Autònoma de Barcelona (UAB), Bellaterra (Cerdanyola del Vallès), Barcelona, Spain

**Keywords:** ACAT1, Breast cancer, CD36, Cholesteryl ester, LDL receptor, SCARB1

## Abstract

**Background:**

The metabolic effect of intratumor cholesteryl ester (CE) in breast cancer remains poorly understood. The objective was to analyze the relationship between intratumor CE content and clinicopathological variables in human breast carcinomas.

**Methods:**

We classified 30 breast carcinoma samples into three subgroups: 10 luminal-A tumors (ER+/PR+/Her2-), 10 Her-2 tumors (ER-/PR-/Her2+), and 10 triple negative (TN) tumors (ER-/PR-/Her2-). We analyzed intratumor neutral CE, free cholesterol (FC) and triglyceride (TG) content by thin layer chromatography after lipid extraction. RNA and protein levels of lipid metabolism and invasion mediators were analyzed by real time PCR and Western blot analysis.

**Results:**

Group-wise comparisons, linear regression and logistic regression models showed a close association between CE-rich tumors and higher histologic grade, Ki-67 and tumor necrosis. CE-rich tumors displayed higher mRNA and protein levels of low-density lipoprotein receptor (LDLR) and scavenger receptor class B member 1 (SCARB1). An increased expression of acetyl-Coenzyme A acetyltransferase 1 (ACAT1) in CE-rich tumors was also reported.

**Conclusions:**

Intratumor CE accumulation is intimately linked to proliferation and aggressive potential of breast cancer tumors. Our data support the link between intratumor CE content and poor clinical outcome and open the door to new antitumor interventions.

**Electronic supplementary material:**

The online version of this article (doi:10.1186/s12885-015-1469-5) contains supplementary material, which is available to authorized users.

## Background

The homeostasis of lipids is deregulated in several tumors [1, 2] including breast cancer [3, 4]. The risk of breast cancer increases in association with high fat diets, overweight, obesity, and type 2 diabetes, conditions characterized by an anomalous lipid profile [5–7]. Plasma levels of total cholesterol, low-density lipoprotein (LDL) and triglycerides (TG) are frequently higher, and high density lipoproteins (HDL) lower, in patients with breast cancer [8, 9]. The expression of lipid metabolism genes is also changed in breast tumors compared to the normal adjacent tissue [10]. Dyslipidemia favors mammary tumor growth and metastasis in *in vivo* models [11], and LDL and HDL stimulate proliferation and migration in *in vitro* breast tumor cell models [12–15]. Taken together, these results show a close relationship between the deregulation of lipid homeostasis and breast cancer.

Plasma lipoproteins are a source of fatty acids (FA) and cholesteryl esters (CE) for tumor cells. FA oxidation is the main source of energy for prostate and pancreatic tumors [16, 17]. Availability of intratumoral CE reduces *de novo* lipid synthesis, favors membrane biogenesis, induces lipid raft formation and alters tumor cell signaling, essential processes for tumor proliferation, invasiveness and survival [18–20]. In concordance with these data, the inhibition of CE synthesis has anticancer effects [21, 22].

Breast tumor subtypes represent different molecular entities that show great heterogeneity in their tumorigenesis, aggressiveness and malignancy and a significant disparity in clinical outcomes and management [23–27]. Furthermore, most investigations about deregulated lipid metabolism in breast neoplasms have been performed *in vitro* and animal models rather than in human samples. This makes it difficult to extrapolate results for clinical practice. Complementary knowledge based on translational approaches is thus required to improve diagnosis and treatment of breast cancer. We hypothesized that intratumor CE levels are associated with clinicopathological variables in human breast carcinomas. The objective of this study was thus to analyze the relationship between intratumor CE content, lipid metabolism mediators, invasion markers, and clinicopathological parameters.

## Material and Methods

### Patient and tumor samples

The clinical-pathological features of patients and tumor samples are summarized in Table 1. Thirty tumor samples were selected retrospectively (from May 2006 to November 2012) from the Tumor Tissue Bank in the Pathology Department at Hospital Santa Creu i Sant Pau, Barcelona, Spain. Tumor samples were classified in three subgroups: 10 cases were Luminal-A tumors (ER+/PR+/Her2-), 10 Her-2 tumors (ER-/PR-/Her2+) and 10 TN tumors (ER-/PR-/Her2-). For this comparative clinicopathological and molecular study, we selected a similar number of each subtype of breast carcinomas. Exclusion criteria were patients with mutations in BRCA1 and BRCA2 genes. Patients were staged according to the TNM staging system and tumors were processed and studied according to the standard protocols [28, 29]. The Ki-67 index was also evaluated by immunohistochemistry. Patients’ medical history and clinical evaluation were fully reviewed. The presence of dyslipidemia and menopause was based on explicit diagnosis in the medical history, assuming that, to diagnose the condition, clinicians have followed the criteria admitted to each time. Patients were treated according to our institution guidelines, essentially first treatment was surgery for patients with stages I and II, followed by adjuvant chemotherapy, endocrine therapy and radiotherapy when indicated.

**Table 1:** Clinical and pathological characteristics of patients and tumor samples

Variables	N = 30
Age (years)	62.5 (48.8-76.0)
***Clinicopathological parameters***	
Breast carcinoma type	
Luminal-A (%)	10 (33)
Her-2 (%)	10 (33)
TN (%)	10 (33)
Histologic types	
Invasive ductal carcinoma (%)	26 (87)
Medullary carcinoma (%)	2 (7)
Papillary carcinoma (%)	1 (3)
Mixed carcinoma (%)	1 (3)
Nottingham combined histologic grade	
Grade I (%)	6 (20)
Grade II (%)	4 (13)
Grade III (%)	20 (67)
TNM staging	
Stage IA (%)	7 (23)
Stage IIA (%)	14 (47)
Stage IIB (%)	4 (13)
Stage IIIA (%)	2 (7)
Stage IIIB (%)	1 (3)
Stage IV (%)	1 (3)
Unknown (%)	1 (3)
Tumor size (cm)	3.00 (2.00-4.00)
Lymph node affected (%)	10 (33)
Vascular invasion (%)	4 (13)
Tumor necrosis (%)	12 (40)
Mean Ki-67 (%)	20 (5–53)
Ki-67 over 20 (%)	15 (50)
***Other conditions***	
Dyslipidemia (%)	11 (38)
Menopause (%)	15 (50)
***Intratumor lipid parameters***	
CE (μg/mg protein)	3.14 (1.45-4.65)
TG (μg/mg protein)	5.91 (2.87-13.95)
Free Chl (μg/mg protein)	4.97 (4.47-5.50)

Data are presented as medians (interquartile ranges) for continuous variables and as frequencies (percentages) for categorical variables

CE: Cholesteryl Esters; Free Chl: Free Cholesterol; Her-2: Human Epidermal Growth Factor Receptor 2; TG: Triglycerides; TN: Triple Negative

This study was conducted according to the Declaration of Helsinki principles, with approval from the Clinical Research Ethics Committee at Institut d’Investigacions Biomèdiques Sant Pau. Written informed consent was obtained from all patients

### Tumor sample collection

Tumor tissue samples were obtained from surgical specimens, mastectomy or lumpectomy, rapidly embedded in OCT (Tissue-Tek, Sakura, Europe, Alphen den Rijn, The Netherlands) and frozen using a histobath (Thermo Shandon, Pittsburgh, PA, USA). A macrodisecction of the samples was performed to remove fatty tissue and exclusively collect tumoral tissue. Immunohistochemistry was carried out using the EnVision Flex™ detection system (Dako/Agilent Technologies; Carpinteria, CA), following the manufacturer´s instructions.

### RNA extraction and cDNA synthesis

Total RNA was extracted from frozen tumoral tissue using TriPure isolation reagent (Roche Molecular Biochemicals) and the RNeasy mini kit (Qiagen, Hilden, Germany) according to the manufacturer’s instructions. Extracted RNA was eluted in 25 μL of nucleases-free water. RNA yield and quality were assessed by agarose electrophoresis and spectrophotometry, and then stored at −80 °C until use. RNA was digested with DNase I (Invitrogen). One μg of total RNA was used for cDNA synthesis according to the protocol provided with the High Capacity cDNA Reverse Transcription kit (Applied Biosystems, Foster City, CA, USA). Recombinant RNasin Ribonuclease Inhibitor (Applied Biosystems) was added to prevent RNase-mediated degradation. The cDNA was also stored at –20C.

### Gene expression analyses by RT-PCR

The expression of different genes involved in lipid metabolism and tumor invasion was analyzed at mRNA level by quantitative real-time reverse transcriptase-polymerase chain reaction (q-RT-PCR). Specific primer and fluorescent TaqMan probes were selected within a list of predesigned assays (Table 2). *18srRNA* (4319413E) was used as a housekeeping gene. We mixed 5 μl of single-stranded cDNA (equivalent to 100 ng of total RNA) with 1 μl of 20x TaqMan Gene Expression Assays for each Assay-on-Demand, 10 μl of TaqMan Universal PCR Master Mix, and 4 μl of nucleases-free water. After gentle mixing, the mixture was transferred into a real-time PCR microplate. The Real-time PCR microplate was sealed, centrifuged, and placed in the sample block of an Applied Biosystems 7300 Real Time PCR System (Applied Biosystems). The thermal cycling conditions were 2 min at 50 °C and 10 min at 95 °C, followed by 40 cycles of 15 s at 95 °C and 1 min at 60 °C. Expression levels were measured in triplicate. The threshold cycle (Ct) values were normalized to the housekeeping gene.

**Table 2:** Mediators of tumor lipid homeostasis and invasion

Marker	Abbreviation	Probe ID
*Tumor Lipid Homeostasis*		
***Lipid internalization***		
Low density lipoprotein receptor-related protein 1	LRP1	Hs00233899_m1
Cluster of Differentiation 36	CD36	
Very low-density lipoprotein receptor	VLDLR	Hs01045922_m1
Low-density lipoprotein receptor	LDLR	Hs00181192_m1
Scavenger receptor class B member 1	*Gene:*SCARB1	
	*Protein:* SR-BI	
***Lipid efflux***		
ATP-binding cassette transporter	ABCA1	Hs01059118_m1
***Cholesterol synthesis***		
3-hydroxy-3-methyl-glutaryl-CoA reductase	HMG-CoAR	Hs00168352_m1
***Fatty acid synthesis***		
Fatty acid synthase	FASN	Hs01005622_m1
***Cholesterol esterification***		
Acetyl-Coenzyme A acetyltransferase 1	ACAT1	Hs00608002_m1
***Cholesterol management***		
Caveolin-1	CAV1	Hs00971716_m1
Liver X receptor α	LXR-α	Hs00172885_m1
Sterol regulatory element-binding transcription factor 1	SREBP1	Hs00231674_m1
Sterol regulatory element-binding transcription factor 2	SREBP2	Hs00190237_m1
*Tumor Invasion*		
Matrix metalloproteinase-2	MMP-2	Hs00234422_m1
Matrix metalloproteinase-9	MMP-9	Hs00234579_m1
Tissue inhibitor of metalloproteinase 1	TIMP1	Hs01092511_m1
Cathepsin S	CTSS	Hs00175407_m1

### Western blotting

Total protein was extracted from tumoral frozen tissue using TriPure isolation reagent (Roche Molecular Biochemicals). Proteins were analyzed by Western blot analysis under non-reducing (LRP1 and VLDLR) and reduced (LDLR, SR-BI, CTSS and MMP9) conditions on polyacrylamide gels for SDS-PAGE. The samples were electrotransferred to nitrocellulose, and the membranes were saturated at room temperature for 1 hour in TTBS (20 mM Tris–HCl pH 7.5, 500 mM NaCl, 0.01 % Tween 20, and 5 % non-fat milk). Western blot analysis was performed with specific monoclonal antibodies against LRP1 (10R-L107c, Fitzgerald), VLDLR (sc-18824, Santa Cruz Biotechnology), LDLR (ab52818, Abcam), SR-BI (sc-67098, Santa Cruz Biotechnology), CTSS (sc-271619, Santa Cruz Biotechnology), and MMP9 (AB805, Chemicon International). Equal protein loading in each lane was verified by staining filters with Pounceau. A reference sample was applied in all blots and the value of the reference sample was used to normalize the other samples of the same blot.

### Lipid extraction and semi-quantitative analysis of cholesteryl ester, free cholesterol and triglyceride content of tumor samples

An aliquot of the frozen tumoral tissue was homogenized in NaOH 0.1 M, and CE, TG and free cholesterol (FC) were partitioned by thin layer chromatography after lipid extraction as previously described [30, 31]. The different concentrations of standards (a mixture of cholesterol, cholesterol palmitate and triglycerides) were applied to each plate. The chromatographic developing solution was heptane/diethylether/acetic acid (74:21:4, vol/vol/vol). The spots corresponding to CE, TG and FC were quantified by densitometry against the standard curve of cholesterol palmitate, triglycerides and cholesterol, respectively, using a computing densitometer.

### Statistical analysis

Statistical analysis was performed using SPSS 15.0 statistical software (SPSS Inc, Chicago, IL). Descriptive statistics were used to characterize the study population and to describe the clinicopathological variables. Data are presented as frequencies (percentages) for categorical variables, and medians (interquartile ranges) for continuous variables. Categorical variables were compared between groups using Pearson´s Chi square test or Fisher’s exact test as required. Continuous variables were compared between groups with the Krusall-Wallis test or non-parametric Mann–Whitney *U* test for medians, as required. In comparisons where differences between groups were detected with the Krusall-Wallis test, the Mann–Whitney *U* test for medians was used with Bonferroni correction to test for differences in pairs (0.05/3 = 0.017). Correlations were performed with Spearman's rank test. Linear regression analysis was performed to explore the association between Ki-67 levels and intratumor CE content. Several models were developed: unadjusted (model 1) and adjusted by age (model 2). Logistic regressions were performed to examine the associations between categorical clinicopathological parameters and intratumor CE content. Model 1 was unadjusted. To establish whether the observed association could be influenced by possible confounders, model 2 was adjusted by age and neoadjuvant therapy. Differences were considered statistically significant when p < 0.05.

## RESULTS

### Clinicopathological findings according to receptor status

Table 3 summarizes clinicopathological characteristics according to receptor status. Her-2 and TN tumors were the most prevalent in grade III and Ki-67 over 20 %. Tumor necrosis was more prevalent in Her-2 and TN than in Luminal-A tumors but did not reach statistical significance (*p* = 0.054). There were no differences in TNM stage, tumor size, number of lymph nodes affected and vascular invasion markers between groups. Concerning other conditions, dyslipidemia was more prevalent in patients with TN carcinomas than in those with Luminal-A or Her-2 tumors.

**Table 3:** Clinical and pathological characteristics according to breast carcinoma type

Variables	Luminal-A	Her-2	TN	
	N = 10	N = 10	N = 10	p-value
Age (years)	66.5 (46.8-85.3)	54.0 (49.8-63.0)	68.5 (50.8-76.0)	0.477
***Clinicopathological parameters***				
Nottingham combined histologic grade				<0.001*
Grade I (%)	5 (50)	0 (0)	1 (10)	
Grade II (%)	4 (40)	0 (0)	0 (0)	
Grade III (%)	1 (10)	10 (100)	9 (90)	
TNM Stage				0.779
Stage I-II (%)	9 (90)	8 (80)	8 (80)	
Stage III-IV (%)	1 (10)	1 (10)	2 (20)	
Unknown (%)	0 (10)	1 (10)	0 (10)	
Tumor size (cm)	2.25 (1.88-3.62)	2.75 (1.73-4.12)	3.25 (2.57-4.25)	0.349
Lymph node affected (%)	4 (40)	3 (30)	3 (30)	0.861
Vascular invasion (%)	1 (10)	2 (20)	1 (10)	0.749
Tumor necrosis (%)	1 (10)	6 (60)	5 (50)	0.054
Ki-67 (%)	5.00 (4.50-7.75)	30.00 (12.50-42.50)^a^	65.00 (21.25-82.50) ^b^	0.001*
Ki-67 over 20 (%)	0 (0)	7 (70)	8 (80)	0.001*
***Other conditions***				
Dyslipidemia (%)	2 (20)	1 (10)	8 (80)	0.003*
Menopause (%)	5 (50)	6 (60)	4 (40)	0.537

Data are presented as medians (interquartile ranges) for continuous variables and as frequencies (percentages) for categorical variables

p-values by Krusall-Wallis test, Pearson´s Chi square test or Fisher’s exact test when corresponding

*Statistically significant

For continuous variables U Mann–Whitney test was performed when Krusall-Wallis test was significant: ^a^Luminal-A*vs.*Her-2; ^b^Luminal-A*vs.* TN; ^c^ Her-2*vs.* TN

CE: Cholesteryl Esters; Free Chl: Free Cholesterol; Her-2: Human Epidermal Growth Factor Receptor 2; TG: Triglycerides; TN: Triple Negative

**Fig. 1:**
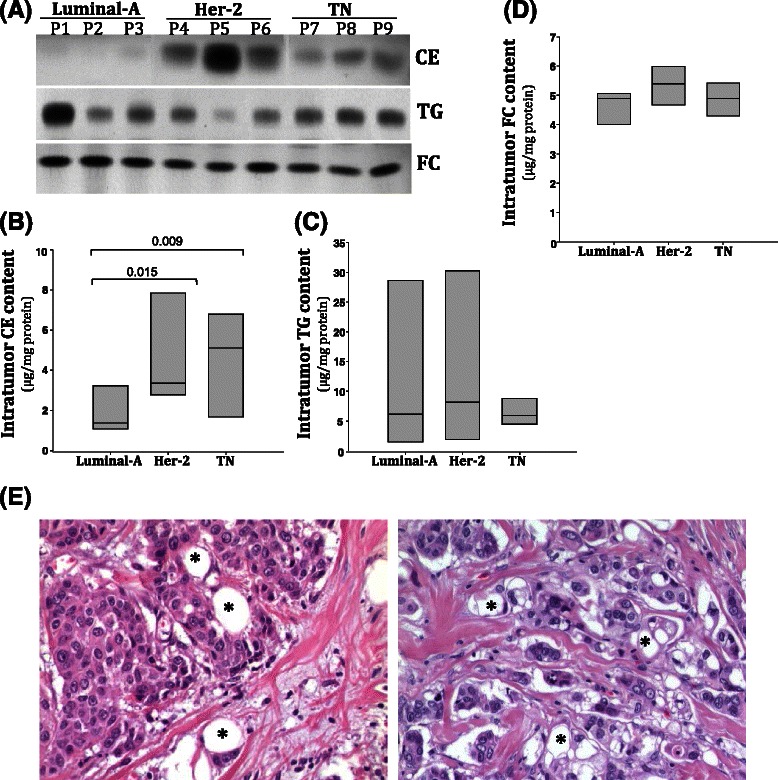
Intratumor cholesteryl ester (CE), triglyceride (TG) and free cholesterol content (FC) in Luminal-A, Her-2 and TN breast tumors. (**a**) Representative thin layer chromatography showing CE, TG and FC bands in three luminal-A, Her-2 and TN representative tumors. Bar graphs show medians (interquartile ranges) of intratumor CE (**b**), TG (**c**) and FC (**d**) content in luminal-A (n = 10), Her-2 (n = 10) and TN (n = 10) groups. (**e**), Representative images showing a high content of intratumor vacuoles (asterisk) in Her-2 and TN breast tumors

Thin layer chromatography after lipid extraction showed that intratumoral CE accumulation was significantly higher in Her-2 and TN tumors than in Luminal-A tumors: Her-2: 3.55 (2.78-7.85) & TN: 5.11 (1.69-6.79 *vs*. Luminal-A: 1.38 (1.09-3.24) (Fig. 1a and b). In contrast, there were no differences in intratumor TG (Fig. 1a and c) or FC (Fig. 1a and d) content between groups. Immunhistochemical studies showed the presence of cytoplasmic vacuoles (asterisks) in Her-2 and TN breast tumors cells (Fig. 1e).

### Molecular analysis according to receptor status

We analyzed the expression of: a) genes involved in lipid metabolism including lipid uptake, cholesterol synthesis, fatty acid synthesis, cholesterol esterification, cholesterol management and cholesterol efflux; and b) genes involved in tumor invasiveness (Table 2). Real time PCR results showed that LDLR and ACAT1 expression was significantly higher in Her-2 than in luminal-A tumors (Additional file 1: Table S1). No differences between tumor subtypes were observed in other mediators of lipid uptake, cholesterol synthesis, fatty acid synthesis, CE synthesis, cholesterol management, or cholesterol efflux. No differences were reported in markers of invasion.

### Clinicopathological findings according to intratumor CE content

To explore the associations between intratumor CE levels and tumor malignancy, the population was divided into statistical tertiles of intratumor CE content: tertile 1: 0.16-2.59 (μg/mg protein), tertile 2: 2.60-5.16 (μg/mg protein), and tertile 3: 5.17-9.92 (μg/mg protein). Tumor samples in tertile 3 were considered the CE-rich group. The remaining tissue samples were included in the control CE group. Table 4 summarizes clinicopathological characteristics according to intratumor CE levels. Regarding receptor status, the highest CE tertile was exclusively composed of Her-2 (40 %) and TN (60 %) carcinomas. Luminal-A tumors were all included in the group with lower CE content. According to histological grade, all tumors in the highest tertile of CE content were grade III. Tumor necrosis and Ki-67 were higher in the CE-rich group than in the control group (80 % vs 20 % and 90 % vs 30 %). Ki-67 levels were higher in tumors of tertile 3 than in tumors of tertiles 1 and 2. There were no statistically significant differences between groups in TNM stage, tumor size, number of lymph nodes affected or vascular invasion markers. There were no differences according dyslipidemia or menopause status between groups. There were no differences in TG or free cholesterol levels between the CE-rich group and other groups.

**Table 4:** Clinical and pathological characteristics according to intratumor cholesteryl esters content tertile

Variables	Control N = 20	CE-rich N = 10	p-value
Age (years)	65.5 (49.8-78.3)	53 (45.5-73.0)	0.267
***Clinicopathological parameters***			
Breast carcinoma type			0.015*
Luminal-A (%)	10 (50)	0 (0)	
Her-2 (%)	6 (30)	4 (40)	
TN (%)	4 (20)	6 (60)	
Nottingham combined histologic grade			0.024*
Grade I (%)	6 (30)	0 (0)	
Grade II (%)	4 (20)	0 (0)	
Grade III (%)	10 (50)	10 (100)	
TNM Stage			0.636
Stage I-II (%)	17 (85)	8 (80)	
Stage III-IV (%)	3 (15)	1 (10)	
Unknown (%)	0 (10)	1 (10)	
Tumor size (units)	2.40 (1.85-3.87)	3.25 (2.57-4.00)	0.231
Lymph node affected (%)	7 (35)	3 (30)	0.560
Vascular invasion (%)	2 (10)	2 (20)	0.407
Tumor necrosis (%)	4 (20)	8 (80)	0.003*
Ki-67 (%)	8.50 (5.00-28.75)	65.00 (37.50-82.50)	<0.001*
Ki-67 over 20 (%)	6 (30)	9 (90)	0.003*
***Other conditions***			
Dyslipidemia (%)	7 (35)	4 (40)	0.466
Menopause(%)	11 (55)	4 (40)	0.603
***Intratumor lipid parameters***			
CE (μg/mg protein)	2.59 (1.29-3.30)	7.30 (5.94-8.67)	<0.001*
TG (μg/mg protein)	4.31 (2.07-18.70)	7.36-14.38)	0.502
Free Chl (μg/mg protein)	4.89 (4.24-5.25)	5.18 (4.71-6.29)	0.155

Data are presented as medians (interquartile ranges) for continuous variables and as frequencies (percentages) for categorical variables

p-values by Mann–Whitney U test or by Fisher’s exact test. *Statistically significant

CE: Cholesteryl Esters; Free Chl: Free Cholesterol; Her-2: Human Epidermal Growth Factor Receptor 2; TG: Triglycerides; TN: Triple Negative

To further explore the relationships between the intratumoral CE accumulation and clinicopathological parameters, we analyzed the association between intratumoral CE accumulation and the clinicopathological categorical variables: histologic grade (III *vs.* I-II), TNM stage (III-IV *vs.* I-II), tumor size > 3 cm, lymph node affected, vascular invasion, tumor necrosis and Ki-67 > 20 %. Logistic regression models showed a direct association between intratumoral CE accumulation and higher histologic grade, tumor necrosis and Ki-67 > 20 % (Tables 5 and 6). To analyze the influence of age on the associations between the intratumor CE content and clinicopathological parameters, logistic regressions were adjusted for these covariables and the association between CE levels and histologic grade, tumor necrosis and Ki-67 > 20 %, remained statistically significant (Table 5). No associations between intratumor CE content and TNM Stage, tumor size, lymph node affected or vascular invasion were reported.

**Table 5:** Association between intratumor cholesteryl ester content and Ki-67

	B (95 % CI)	β	*p* value
**Model 1**			
CE	7.323 (3.981-10-666)	0.662	<0.001*
**Model 2**			
CE + Age	7.353 (3.731-10.975)	0.665	<0.001*

B: Beta; CI: Confidence Interval; β: Standardized betas

Model 1: Unadjusted; Model 2: Adjusted by age

*Statically significant

CE: Cholesteryl Esters

**Table 6:** Association between intratumor cholesteryl esters content and clinicopathological parameters

	Model 1		Model 2	
	OR (95 % CI)	*p-*value	OR (95 % CI)	*p-*value
Histologic grade (III *vs*. I-II)	2.115 (1.134-3.945)	0.018*	2.084 (1.092-3.978)	0.037*
TNM Stage (III-IV *vs.* I-II)	0.693 (0.332-1.447)	0.329	0.675 (0.282-1.617)	0.378
Tumor size >3 cm (%)	1.056 (0.794-1.404)	0.709	0.999 (0.716-1.394)	0.997
Lymph node affected	0.811 (0.569-1.158)	0.249	0.808 (0.561-1.164)	0.808
Vascular invasion	1.112 (0.723-1.711)	0.629	1.098 (0.715-1.685)	0.670
Tumor necrosis	1.718 (1.123-2.630)	0.013*	1.906 (1.118-3.249)	0.018*
Ki-67 over 20 %	1.781 (1.120-2.831)	0.015*	1.746 (1.078-2.829)	0.024*

OR: Odds Ratio. CI: Confidence Interval

Model 1: Unadjusted; Model 2: Adjusted by age and neoadjuvant therapy

*Statically significant

### Molecular analysis according to intratumor CE content

**Fig. 2:**
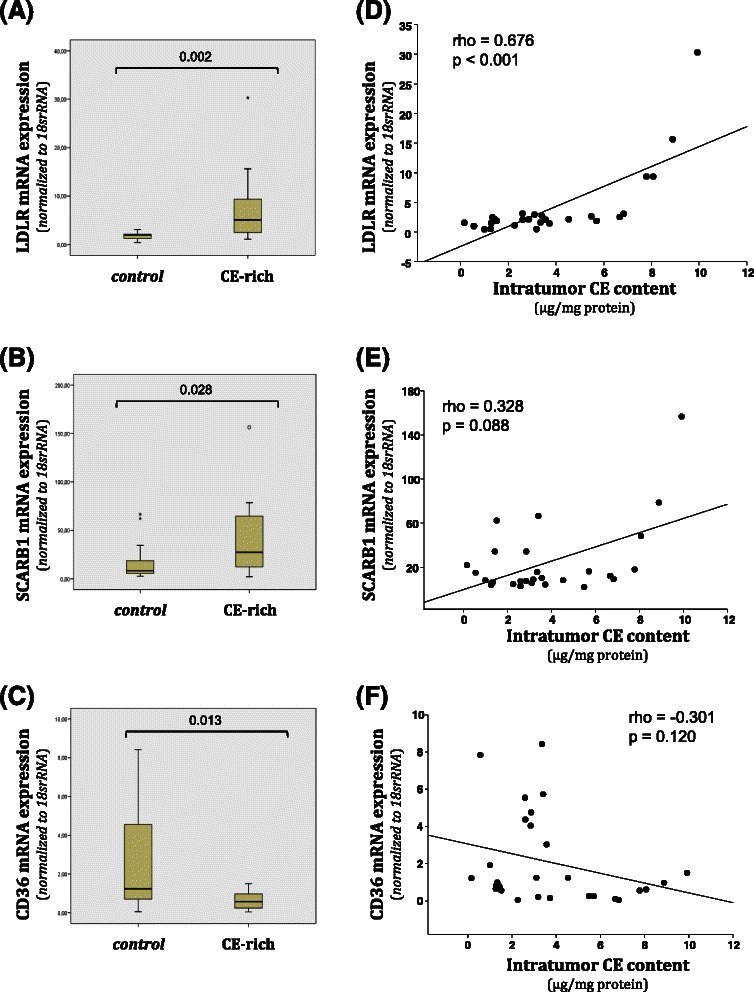
Analysis of LDLR, SCARB1 and CD36 mRNA expression in control and CE-rich groups and correlation with intratumor CE-content. Real-time PCR analysis of LDLR, SCARB1 and CD36 mRNA expression. Data were processed by especially designed software based on the Ct value of each sample and normalized to *18srRNA.* Bar graphs showing medians (interquartile ranges) of normalized values of LDLR (**a**), SCARB1 (**b**) and CD36 (**c**) mRNA expression in control (N = 20) and CE-rich (N = 10) groups. Correlations between intratumor CE content and LDLR (**d**), SCARB1 (**e**) and CD36 (**f**) mRNA expression in breast tumors

**Fig. 3:**
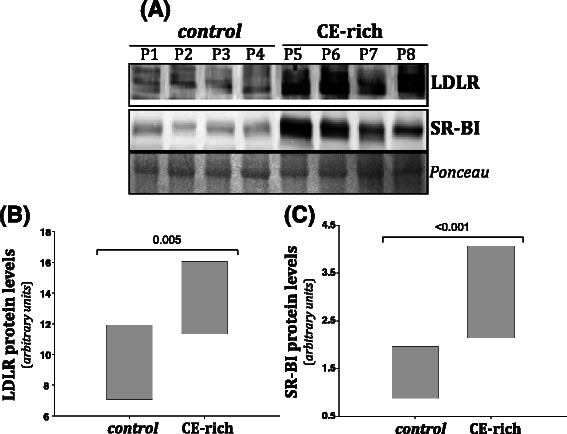
Analysis of LDLR and SR-BI protein levels in control and CE-rich groups. Representative Western blot analysis showing LDLR and SR-BI protein levels in breast tumors (**a**). Ponceau staining was used as loading control. Bar graphs showing medians (interquartile ranges) of normalized LDLR (**b**) and SCARB1 (**c**) protein levels in control (N = 20) and CE-rich (N = 10) groups

**Fig. 4:**
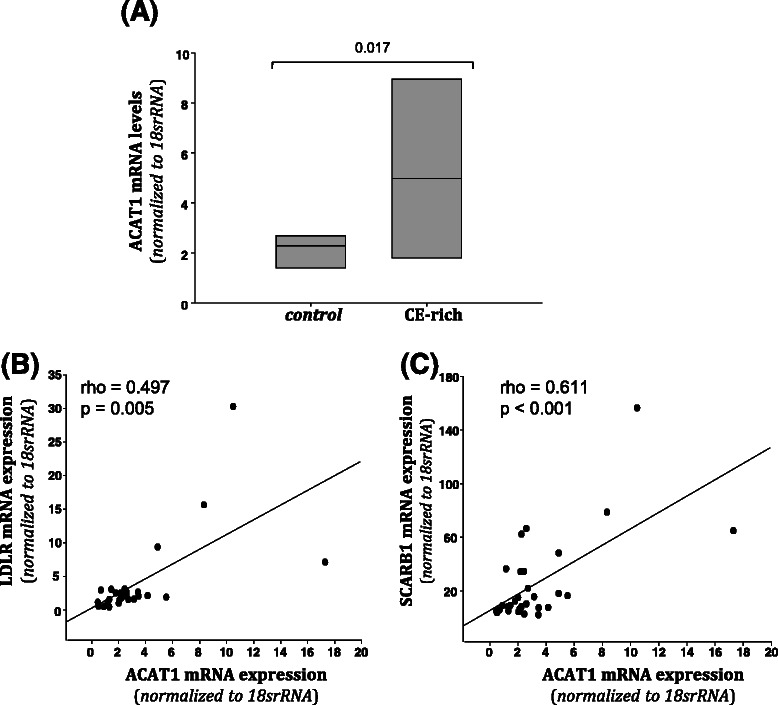
Analysis of ACAT mRNA expression in control and CE-rich groups and its correlation with intratumor CE-content. Real-time PCR analysis of ACAT mRNA expression (**a**). Data were processed using a specially designed software based on Ct value of each sample and normalized to *18srRNA.* Bar graphs showing medians (interquartile ranges) of normalized values of ACAT mRNA expression in control (N = 20) and CE-rich (N = 10) groups. Correlations between ACAT and LDLR mRNA expression (**b**) and between ACAT and SCARB1 (**c**) mRNA expression in breast tumors

CE-rich tumors presented higher LDLR (Fig. 2a) and SCARB1 (Fig. 2b) mRNA expression than controls. In contrast, CD36 mRNA expression was downregulated in CE-rich tumors compared to controls (Fig. 2c). A statistically significant correlation between LDLR expression levels and intratumor CE content was observed (Fig. 2d). The correlation between intratumor CE content and LDLR (positive) (Fig. 2e) or CD36 (negative) (Fig. 2f) did not reach statistical significance. In line with real time PCR analysis, western blot analysis showed that protein levels or LDLR (Fig. 3a and b) and SCARB1 (Fig. 3a and c) were significantly higher in CE-rich breast tumors than in controls. Real time PCR experiments also showed higher ACAT1 mRNA expression levels in CE-rich tumors than in the control group (Fig. 4a). ACAT mRNA expression was positively correlated with LDLR (Fig. 4b) and SCARB1 (Fig. 4c) mRNA expression. There were no differences in the expression of lipid mediators implicated in cholesterol synthesis (HMG-CoAR), cholesterol management (CAV1, LXR-α, SREBP1 and SREBP2) and cholesterol efflux (ABCA1) between groups. No differences were observed in invasiveness markers (MMP2, MMP9, TIMP and CTSS) between groups (Additional file 1: Table S2).

## Discussion

This investigation demonstrated an important association of intratumor cholesteryl ester (CE) accumulation with tumor proliferation but not with invasiveness. We also found that mechanisms potentially involved in intratumoral CE accumulation may be lipid internalization through the overexpression of LDLR and SCARB1 receptors and cholesterol esterification through the upregulation of ACAT1 enzyme.

Group-wise comparisons, linear regression and logistic regression models showed that human breast tumors in the highest tertile of CE content, CE-rich tumors, were intimately associated with higher breast tumor aggressive potential. Our results also showed a close relationship between intratumoral CE accumulation and Ki-67, a well-known marker of proliferation, associated with shorter disease-free survival and overall survival and more frequent tumor recurrence [32]. In addition, we found that intratumoral CE accumulation was intimately associated with higher tumor necrosis, an independent prognostic variable of progression-free and cancer-specific survival [33]. Our data support a close link between breast intratumor CE accumulation and tumor proliferation, indicating that intratumor CE accumulation is associated with poor clinical outcome of patients with breast cancer. These results are in line with previous findings showing an association between intratumor neutral lipids and tumor malignancy [20, 34]. At present we could only speculate about the role of CE in tumor proliferation and aggressive potential; however, it has been proposed that stored CE may reduce the energetically costly lipid synthesis, increase membrane synthesis and rigidity, and induce a pro-tumorigenic signaling, all mechanisms linked to the malignancy of breast cancer [2, 18, 20].

Results for the present study showed a tight correlation between intratumor CE accumulation and LDLR expression and that both LDLR mRNA and protein levels were significantly elevated in CE-rich breast carcinomas. These results suggest that LDLR overexpression facilitates an exacerbated LDL-cholesterol uptake by breast tumor cells. A crucial question is why LDLR, a receptor that is usually downregulated by intracellular cholesterol [35], remains upregulated despite elevated intratumor CE levels. It has been proposed that increased ACAT activity, may contribute to cholesterol removal from endoplasmic reticulum by promoting the cholesterol esterification rate [36]. Cholesterol of the endoplasmic reticulum represses the activation of SREBPs, key transcription factors involved in the positive modulation of LDLR and other lipogenic genes such as HMG-CoA reductase. Our results show that the expression of SREBP-1, SREBP-2, LDLR or HMG-CoA reductase was not significantly reduced in breast CE-rich tumors in which ACAT was upregulated. These results indicate that intracellular cholesterol could not repress SREBP or SREBP-modulated genes because cholesterol is rapidly esterified through ACAT in tumor cells. Consistently, we found a close and direct correlation between ACAT and LDLR overexpression in breast tumors. Therefore, as previously suggested in breast cancer cell lines [36], overexpression of ACAT in human breast cancer maintains low endoplasmic reticulum cholesterol levels, thereby allowing LDL cholesterol to enter the tumor. Our results show that, both LDLR and ACAT overexpression contribute to tumor malignancy in breast cancer patients by promoting intratumoral CE accumulation. Our data are in agreement with previous *in vitro* studies highlighting the pivotal role of LDLR [36], and ACAT [19, 37] overexpression in massive cholesterol internalization, in particular in ER- breast cell lines. Here, we also show a significant upregulation of another lipoprotein receptor, SCARB1, in human CE-rich breast carcinomas. SCARB1 is a cell-surface glycoprotein that mediates LDL-CE [38], and HDL-CE [39], selective uptake. According to our results, SCARB1 levels, similar to LDLR levels, are significantly elevated in CE-rich breast carcinomas and significantly correlated with ACAT expression. Moreover, SCARB1 has been reportedly upregulated by hypercholesterolemia in a breast cancer mice model [40]. Taken together, these results suggest that SCARB1, besides LDLR, may actively contribute to human breast tumor CE accumulation and aggressive potential. In line with this, it has been reported that SR-BI knockdown inhibits cellular proliferation in *in vitro* [10], and *in vivo* breast cancer models [18]. These results suggest that LDLR and SCARB1 overexpression combined with ACAT overexpression are key mechanisms for intratumor CE accumulation in human breast carcinomas.

Differently to LDLR and SCARB1 upregulation, CD36 mRNA expression was significantly downregulated in more aggressive CE-rich breast cancer tumors. This is an interesting finding since the loss of CD36 has been proposed as one of the early events in breast carcinogenesis [41]. CD36 is lower in high density breast tissue than in normal density tissue [42]. Notably, normal adjacent tissue from TN tumors exhibited significantly lower CD36 expression levels than ER+ or Her-2 tumors, suggesting that low expression levels of CD36 may predispose to TN tumors and to poor disease outcomes [42]. Despite the capacity of CD36 to bind native and modified lipoproteins [43], CD36 does not seem to contribute to intratumor CE accumulation in breast cancer. Further studies are required to know why CD36 is downregulated in CE-rich breast cancer carcinomas.

**Fig. 5:**
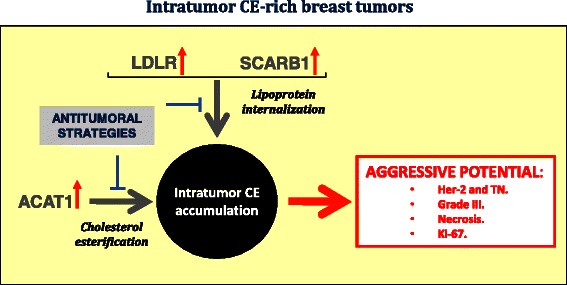
Representative scheme showing potential mechanisms involved in intratumor CE accumulation in breast cancer patients and their relation with breast cancer aggressive potential

Previous *in vitro* studies showed that modified lipoproteins induced breast tumor proliferation, migration and invasiveness [12, 13, 44]. Blockade of CE entry and biosynthesis led to decreased cell migration [18, 36]. In contrast, previous results from our group showed that intracellular CE accumulation decreased human vascular cell migration even in the presence of the hypoxic stimulus [45, 46]. In line with the negative effect of intracellular CE content on vascular cell migration, we could not detect a relation of intratumoral CE accumulation with migration and invasion markers (MMP9, MMP2, TIMP and CTSS) in breast tumors, nor with clinicopathological characteristics of invasion (TNM stage, number of lymph nodes affected, and vascular invasion). Further investigation is required to explain why intratumor CE do not show association with invasiveness despite CE-rich tumors have a high aggressive potential,

Lipid profiling classifications have the advantage over molecular classifications which allows better stratification of breast cancer malignancy [47]. Our study shows that intratumor CE accumulation is a good indicator of human breast cancer proliferation. We suggest thus that intratumor CE would be a marker of aggressive potential after mastectomy or lumpectomy and that cholesterol internalization and esterification mediators are potential targets to counteract intratumor CE accumulation (summarized in Fig. 5).

A larger study sample would have been desirable and our findings should be reproduced in a significantly larger cohort. A detailed description of cause-effect mechanisms cannot be provided when working with human samples from breast cancer tumors.

## Conclusion

In conclusion, our results highlight intratumor CE accumulation as a potential indicator of proliferation in human breast cancer. LDLR and SCARB1 overexpression besides ACAT1 activation may be crucial players causing intratumoral CE accumulation. These results thus open the door to new anticancer therapeutic strategies.

## Additional file

Additional file 1:
**Expression level of genes involved in lipid metabolism.**
