# Supplementary material for: Intratumor cholesteryl ester accumulation is associated with human breast cancer proliferation and aggressive potential: a molecular and clinicopathological study
Source: BMC Cancer. 2015 Jun 9;15:460. doi: 10.1186/s12885-015-1469-5 (PMC4460760; doi:10.1186/s12885-015-1469-5)
Supplement: Additional file 1: — Expression level of genes involved in lipid metabolism. [file 12885_2015_1469_MOESM1_ESM.docx]

| **Table 1S.** Gene expression according to breast carcinoma type. | | | | | | | | | |
| --- | --- | --- | --- | --- | --- | --- | --- | --- | --- |
|  |  |  | |  | |  | | | |
| **Variables** | **Luminal-A** | | **Her-2** | | **TN** | | |  |  |
|  | **N=10** | | **N=10** | | **N=10** | | | **p-value** |  |
|  |  | |  | |  | | |  |  |
| *mRNA expression* |  | |  | |  | | |  |  |
| LRP1 (a.u) | 4.10 (2.03-13.65) | | 4.47 (1.89-5.56) | | 4.62 (1.02-8.67) | | | 0.682 |  |
| CD36 (a.u) | 0.91 (0.53-4.99) | | 2.26 (0.60-4.66) | | 0.58 (0.10-1.12) | | | 0.063 |  |
| VLDLR (a.u) | 2.23 (0.61-3.57) | | 3.20 (2.09-8.00) | | 2.70 (0.87-9.20) | | | 0.301 |  |
| LDLR (a.u) | 1.53 (0.88-2.24) | | 2.79 (2.06-9.35) ^a^ | | 2.01 (1.11-4.08) | | | 0.046* |  |
| SCARB1 (a.u) | 12.06 (4.99-34.39) | | 9.03 (4.97-25.69) | | 14.35 (8.26-62.91) | | | 0.459 |  |
| ABCA1 (a.u) | 1.29 (0.69-2.95) | | 1.72 (1.25-4.13) | | 2.42 (0.81-3.81) | | | 0.503 |  |
| ACAT1 (a.u) | 2.17 (1.29-2.55) | | 3.54 (2.64-4.98) ^a^ | | 2.10 (1.36-6.31) | | | 0.047* |  |
| CAV1 (a.u) | 2.38 (1.37-7.40) | | 2.27 (1.485.92) | | 3.52 (0.95-8.11) | | | 0.971 |  |
| HMGCoA (a.u) | 4.42 (1.68-6.67) | | 7.60 (4.07-13.77) | | 6.03 (2.52-11.37) | | | 0.298 |  |
| FASN (a.u) | 1.37 (0.36-3.34) | | 0.64 (0.26-4.1) | | 0.28 (0.19-0.56) | | | 0.083 |  |
| LXR-α (a.u) | 18.74 (9.48-21.24) | | 19.01 (6.47-40.82) | | 21.01 (14.29-55.50) | | | 0.696 |  |
| SREBP1 (a.u) | 40.81 (22.41-54.62) | | 29.81 (18.90-57.42) | | 26.71 (1406-48.63) | | | 0.620 |  |
| SREBP2 (a.u) | 12.20 (10.11-18.12) | | 11.63 (10.86-29.84) | | 12.82 (7.32-28.24) | | | 0.960 |  |
| CTSS(a.u) | 1.66 (0.84-8.90) | | 4.30 (0.80-9.70) | | 2.23 (1.34-7.17) | | | 0.851 |  |
| MMP9m(a.u) | 2.66 (0.57-2.98) | | 2.90 (2.43-7.89) | | 3.95 (1.85-8.90) | | | 0.153 |  |
| MMP2 (a.u) | 2.85 (1.21-8.57) | | 3.68 (1.63-7.19) | | 2.36 (1.484.86) | | | 0.708 |  |
| TIMP1 (a.u) | 1.40 (1.15-3.72) | | 1.28 (0.61-3.48) | | 1.23 (0.31-2.02) | | | 0.277 |  |
|  |  | |  | |  | | |  |  |
| *Protein level* |  | |  | |  | | |  |  |
| LRP1 (a.u) | 11.13 (9.83-13.83) | | 10.49 (6.86-14.54) | | 11.69 (8.49-13.51) | | | 0.756 |  |
| VLDLR (a.u) | 8.34 (1.82-18.55) | | 4.42 (0.37-31.16) | | 8.32 (5.37-16.27) | | | 0.836 |  |
| LDLR (a.u) | 8.85 (6.49 -12.51) | | 10.79 (8.16-12.76) | | 12.34 (7.48-15.45) | | | 0.468 |  |
| SR-BI (a.u) | 1.46 (1.01-1.98) | | 1.48 (0.83-2.41) | | 2.45 (1.81-3.87) | | | 0.089 |  |
| CTSS (a.u) | 5.93 (3.22-11.21) | | 16.56 (3.00-19.66) | | 8.86 (4.81-19.79) | | | 0.488 |  |
| MMP9 (a.u) | 12.06 (9.88-15.34) | | 11.49 (5.51-17.36) | | 10.84 (7.17-13.43) | | | 0.656 |  |
|  |  |  | |  | |  |  |  |  |
| Data are presented as medians (interquartile ranges).  p-values by Krusall-Wallis test.  *: Statistically significant  U Mann-Whitney test was performed when Krusall-Wallis test was significant: ^a^ Luminal-A *vs.* Her-2; ^b^ Luminal-A *vs.* TN; ^c^ Her-2 *vs.* TN.  Her-2: Human Epidermal Growth Factor Receptor 2; TN: Triple Negative. | | | | | | |  |  |  |

| **Table 2S.** Gene expression according to intratumorcholesteryl esters content tertile. | | | |
| --- | --- | --- | --- |
|  |  |  |  |
| **Variables** | **Control** | **CE-rich** | **p-value** |
|  | **N=20** | **N=10** |  |
|  |  |  |  |
| *mRNA expression* |  |  |  |
| LRP1 (a.u) | 4.35 (1.85-7.61) | 4.90 (1.45-8.67) | 0.812 |
| VLDLR (a.u) | 2.69 (1.04-7.01) | 2.72 (1.71-4.05) | 0.983 |
| ABCA1 (a.u) | 2.20 (0.77-3.52) | 1.37 (0.99-3.05) | 0.448 |
| CAV1 (a.u) | 2.68 (1.46-6.22) | 3.01 (081-9.55) | 0.746 |
| HMGCoA (a.u) | 5.68 (1.80-8.72) | 6.00 (3.33-11.27) | 0.619 |
| FASN (a.u) | 2.41 (0.31-6.68) | 0.34 (0.24-1.08) | 0.100 |
| LXR-α (a.u) | 19.87 (10.36-26-10) | 15.79 (9.55-55.50) | 0.681 |
| SREBP1 (a.u) | 40.81 (25.10-53.66) | 22.40 (12.60-42.50) | 0.153 |
| SREBP2 (a.u) | 12.20 (10.35-22.97) | 11.62 (7.19-25.37) | 0.729 |
| ADRP (a.u) | 10.56 (5.78-25.90) | 10.56 (5.78-25.91) | 0.835 |
| CatS (a.u) | 2.95 (1.30-7.13) | 2-73 (1.16-7.71) | 0.812 |
| MMP9 (a.u) | 2.84 (2.12-3.20) | 5.14 (1.31-10.01) | 0.328 |
| MMP2 (a.u) | 3.27 (1.57-6.82) | 1.75 (1.14-7.15) | 0.422 |
| TIMP1 (a.u) | 1.30 (0.72-3.40) | 1.49 (0.37-2.45) | 0.650 |
|  |  |  |  |
| *Protein level* |  |  |  |
| LRP1 (a.u) | 10.92 (9.39-12.42) | 11.75 (7.27-14.71) | 0.779 |
| VLDLR (a.u) | 7.45 (2.36-19.55) | 7.32 (0.72-11.63) | 0.559 |
| CTSS (a.u) | 8.83 (3.20-17.86) | 8.95 (4.81-18.72) | 0.713 |
| MMP9 (a.u) | 12.06 (8.72-15.31) | 11.15 (7.03-13.15) | 0.373 |
|  |  |  |  |
| Data are presented as medians (interquartile ranges).  p-values by Mann-Whitney U test. | | | |
